# Multivariate statistical assessment of heavy metal pollution sources of groundwater around a lead and zinc plant

**DOI:** 10.1186/1735-2746-9-29

**Published:** 2012-12-17

**Authors:** Abbas Ali Zamani, Mohammad Reza Yaftian, Abdolhossein Parizanganeh

**Affiliations:** 1 Phase Equilibria Research Laboratory, Department of Chemistry, Faculty of Science, University of Zanjan, Zanjan, Iran; 2 Environmental Science Research Laboratory, Department of Environmental Science, Faculty of Science, University of Zanjan, Zanjan, Iran

**Keywords:** Heavy metals, Groundwater, Differential pulse polarography, Multivariate data analysis

## Abstract

The contamination of groundwater by heavy metal ions around a lead and zinc plant has been studied. As a case study groundwater contamination in Bonab Industrial Estate (Zanjan-Iran) for iron, cobalt, nickel, copper, zinc, cadmium and lead content was investigated using differential pulse polarography (DPP). Although, cobalt, copper and zinc were found correspondingly in 47.8%, 100.0%, and 100.0% of the samples, they did not contain these metals above their maximum contaminant levels (MCLs). Cadmium was detected in 65.2% of the samples and 17.4% of them were polluted by this metal. All samples contained detectable levels of lead and iron with 8.7% and 13.0% of the samples higher than their MCLs. Nickel was also found in 78.3% of the samples, out of which 8.7% were polluted. In general, the results revealed the contamination of groundwater sources in the studied zone. The higher health risks are related to lead, nickel, and cadmium ions. Multivariate statistical techniques were applied for interpreting the experimental data and giving a description for the sources. The data analysis showed correlations and similarities between investigated heavy metals and helps to classify these ion groups. Cluster analysis identified five clusters among the studied heavy metals. Cluster 1 consisted of Pb, Cu, and cluster 3 included Cd, Fe; also each of the elements Zn, Co and Ni was located in groups with single member. The same results were obtained by factor analysis. Statistical investigations revealed that anthropogenic factors and notably lead and zinc plant and pedo-geochemical pollution sources are influencing water quality in the studied area.

## Introduction

Water is one of essential compounds for all forms of plants and animals [1], thus its pollution is generally considered more important than soil and air. Due to its specific centeracteristics, this liquid bears unique properties. It is the most effective dissolving agent, and adsorbs or suspends many different compounds [2].

More than one billion people in the world do not have suitable drinking water, and two to three billions lack access to basic sanitation services. About three to five millions die annually from water related diseases [3].

Surface water (fresh water lakes, rivers, streams) and groundwater (borehole water and well water) are the principal natural water resources. Nowadays one of the most important environmental issues is water contamination [4,5]. Heavy metals are among the major pollutants of water sources [6]. Despite this, heavy metals are sensitive indicators for monitoring changes in the marine environment. Due to human industrial activities, the levels of heavy metals in the aquatic environment are seriously increasing and have created a major global concern [7,8]. Some of these metals are essential for the growth, development and health of living organisms, whereas others are non-essential as they are indestructible and most of them are categorized as toxic species on organisms [9]. Nonetheless the toxicity of metals depends on their concentration levels in the environment. With increasing concentrations in environment and decreasing the capacity of soils towards retaining heavy metals, they leach into groundwater and soil solution. Thus, these toxic metals can be accumulated in living tissues and concentrate through the food chain.

Cadmium is regarded as the most serious contaminant of the modern age [10]. Copper is classified as a priority pollutant because of its adverse health effects [11]. Zinc and iron are essential elements and are generally considered to be non-toxic below certain levels [12]. Lead is not an essential trace element in any organism and has no known biological function. It can cause a variety of harmful health effects [13] and is known as a fatal neurotoxicant [14]. Excessive concentrations of cobalt can cause death and various compounds of nickel are carcinogenic [15]. These menaces provoke the studies on the monitoring of these heavy metals in this chain being important for protection of public health.

A variety of techniques including x-ray fluorescence (XRF), neutron activation analysis (NAA), inductively coupled plasma-atomic emission spectrometry (ICP-AES), atomic absorption spectrometry (AAS) and graphite furnace atomic absorption spectrometry (GFAAS) have been used for evaluating the heavy metal concentration in environmental samples [16-20]. Beside their valuable centeracteristics, these techniques suffer from some disadvantages such as heavy capital cost, expensive maintenance, and insufficient sensitivity for very low concentrations of metals. Voltammetric methods are known as sensitive techniques for determination of a variety of chemical species [21]; among these techniques, differential pulse polarography (DPP) bears some advantages for accurate and precise detection and determination of trace amounts of heavy metal ions in environmental samples [22,23].

Evaluation of the contaminants resulted from excavation of zinc and lead mines and development of related industries in Zanjan province-Iran and their negative environmental impacts is critical and important. Lack of a systematic investigation of the probable heavy metals contamination around National Iranian Lead and Zinc Company (NILZ) in Bonab Industrial Estate (BIE), in Zanjan province, promotes to assess the quality of groundwater sources in this industrial zone. These are the main sources of drinking water and irrigation for a part of people who live around NILZ Company. In this research, DPP technique was used to determine the concentrations of seven heavy metals (iron, cobalt, nickel, copper, zinc, cadmium and lead) in water samples and the results were compared with the maximum contaminant levels (MCLs) specified by WHO as well as Institute of Standards and Industrial Research of Iran (ISIRI). The multivariate statistical analysis was conducted to categorize the metals and to distinguish the source of the contaminants.

## Materials and methods

### Study area

Zanjan province (located in north west Iran), has a large metalliferrous site and has been considered as a traditional mining region since antiquity [24]. There are still large reserves of lead and zinc in the area. Both mines and smelting units within the province present a risk of contamination of soils, plants, and surface/groundwater resources through dissemination of particles carrying metals by wind action and/or by runoff from the tailings [25]. Transportation of concentrated ore by trucks for about 110 kilometers from mines in Angouran to NILZ is another anthropogenic source of metal contamination, especially along the roads.

In this study, Bonab Industrial Estate (BIE) and its neighborhood was selected for detailed study. The research was focused on the environmental impacts of NILZ Company (36° 66′ N, 48° 48′ E) located within BIE, about 12 km east of Zanjan city. The NILZ Company was established in 1992, with a current consumption of about 300,000 tons of raw ore and an annual production of 55000 tons of Pb and Zn [26,27]. The plant is situated over an aquifer, which is the only source of fresh water available in the area, supplying a part of drinking water to Zanjan citizens and its neighboring areas as well as water used for agricultural and industrial consumptions. The tailings from BIE, estimated to be about 2.5 million tons, contain a variety of toxic elements, notably Pb, Zn, and Cd [26]. They are damped in the vicinity of the Estate and are exposed to wind and rain, contributing to soil, surface and groundwater contamination.

### Sample collection and storage

**Figure 1:**
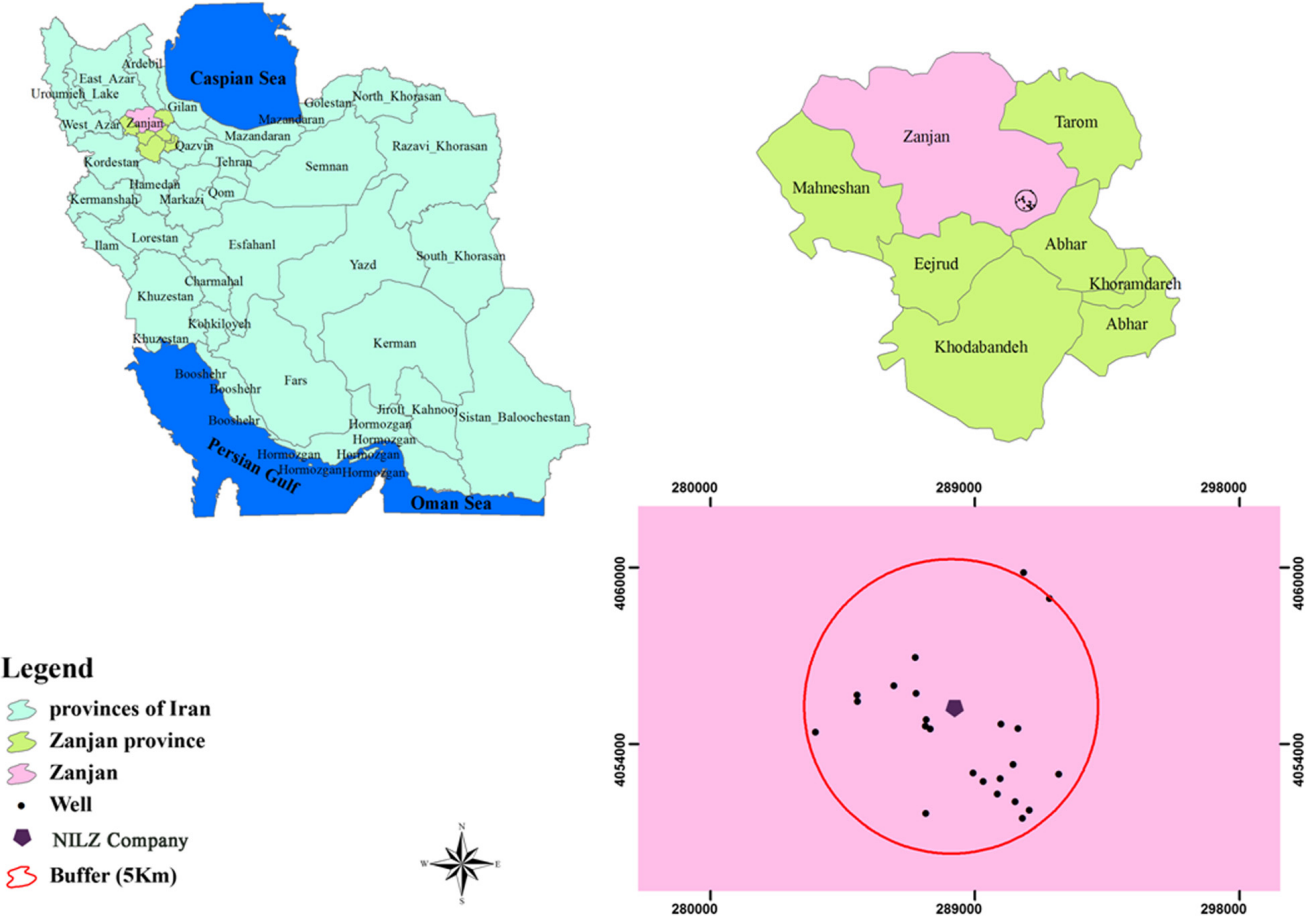
Location map of the studied area indicating sampling points.

To examine the extent of the contamination by toxic metals leached from tailings, 23 spring/groundwater samples were collected and analyzed from the studied area. Nineteen groundwater and four spring water stations were selected from the site within a radius of five km from NILZ Company (Figure 1).

Sampling stations were selected, taking into account the direction of groundwater flow (west), direction of prevailing winds (west and south west) and also the density of the population within the studied area. However, limitations on number and distribution of sampling stations are set due to the spatial distribution of available bore wells within the studied area. Table 1 shows the location of sampling stations for this study.

**Table 1:** GPS location and some physical properties of sampling wells

**Site/location**	**GPS location (UTM)**		**pH**	**EC (μS /cm)**	**DO (mg /L)**	**t (°C)**	**Depth (m)**	**Distance**^**1**^**(m)**
	**X**	**Y**						
W1	290458	4054526	7.81	576	6.91	16	60	2418
W2	289862	4052820	7.93	465	7.46	16	75	2958
W3	290843	4051745	7.58	729	8.36	15	50	4349
W4	289758	4052297	7.70	758	7.73	15	35	3308
W5	287326	4051641	8.15	900	7.10	28	spring	3617
W6	290619	4051470	7.41	1607	7.51	14	13	4512
W7	284992	4055674	7.86	500	6.40	17	45	3234
W8	284999	4055445	8.23	1857	6.95	18	32	3247
W9	283572	4054398	8.27	826	7.22	27	spring	4736
W10	286973	4056943	7.51	361	8.39	17	150	2203
W11	289883	4054679	7.45	369	8.07	19	150	1777
W12	291848	4052965	7.53	480	8.87	15	150	4352
W13	290659	4059821	7.87	334	9.03	16	spring	5320
W14	291539	4058941	8.29	384	7.10	26	spring	5073
W15	290371	4052030	7.52	990	9.36	13	25	3971
W16	289285	4052723	7.45	1157	8.22	14	10	2746
W17	288931	4053010	7.43	1073	7.78	14	150	2344
W18	287475	4054510	7.85	815	9.26	18	20	995
W19	287004	4055727	8.08	564	7.59	14	50	1300
W20	287319	4054582	7.20	326	7.20	15	13	1054
W21	286247	4055985	7.35	850	7.70	15	70	2183
W22	287340	4054826	7.50	671	6.97	15	42	927
W23	290294	4053296	7.80	415	8.90	16	70	2837

^1^ Distance from NILZ Company.

From each station three replicate samples were selected for analysis. Glassware and vessels were treated in 10% (v/v) nitric acid solution for 24 h and were washed with distilled and deionized water. The samples were collected in polypropylene containers, labeled and immediately few drops of HNO_3_ (ultra pure grade) to pH < 2 were added to prevent loss of metals, bacterial and fungal growth and then stored in a refrigerator.

### Reagents and standards

All the chemicals used in this study were mostly reagents of highest grades (Merck) and used without further treatment. The chemicals used were: dimethylglyoxime (>99%), ammonia solution (25%), ammonium chloride (>99.8), acetic acid (>99.8), hydrochloric acid (37%), nitric acid (65%), pyrocatechol (>99%), and sodium hydroxide (>97%). The heavy metal standards were prepared from stock solutions of 1000 ± 5 mg/L (Merck) by successive dilution with ultra-pure water. Polargraphic mercury was used as electrode in heavy metal determination (Merck).

### Sample digestion

Groundwater samples were filtered through 0.45 μm filters. To ensure the removal of organic impurities from the samples and thus preventing interference in analysis, the samples were preserved and digested with concentrated nitric acid. To this end 1 mL of nitric acid was added to water sample in 50 mL volumetric flask.

### Sample analysis in the field

The pH, electrical conductivity (EC) and dissolved oxygen (DO) of the samples were immediately measured at sampling stations by using a portable digital pH meter (Hach HQ 40d). Recorded pH and EC of samples varied in the range of 7.2 -8.3 and 326–1857 (μS /cm) respectively (Table 1). The pH values of the samples were within the WHO range (6.5 -8.5) but those of ECs were below the announced value of MCL by WHO (1500 μS /cm), except for samples number W6 and W8.

### Sample analysis

Water samples were analyzed for the presence of iron, cobalt, nickel, copper, zinc, cadmium and lead using a differential pulse polarography (Metrohm 797 VA). Dissolved air was removed from the solutions by degassing with N_2_ gas (99.999%) for 5–10 min prior to each run. Standard addition method was used for the analysis. The polarography parameters are given in Table 2. Digested samples were analyzed in triplicate and the average concentrations of metals were reported in μg/L.

**Table 2:** Instrument operating parameters for the analysis of the investigated heavy metals

**Parameters**	**Heavy metals**		
	**Fe**^**1**^	**Co and Ni**^**2**^	**Cu, Zn, Cd and Pb**^**3**^
Working electrode	HMDE	HMDE	HMDE
Drop size	7	4	4
Stirrer speed	2000 rpm	2000 rpm	2000 rpm
Mode	DP	DP	DP
Purge time	300 s	300 s	300 s
Deposition potential	−300 mV	−0.7 V	−1.15V
Deposition time	60 s	90 s	90 s
Equilibrium time	5 s	10 s	10 s
Pulse amplitude	50 mV	50 mV	50 mV
Start potential	−200 mV	−0.8 V	−1.15 V
End potential	−550 mV	−1.25 V	0.05 V
Voltage step	4 mV	4 mV	6 mV
Voltage step time	0.4 s	0.3 s	0.1 s
Sweep rate	10 mV/s	13 mV/s	60 mV/s
Peak potential	−380 mV	−1.13, -0.97V	−0.10, -0.98, -0.56, -0.38 V

^1^10 mL sample solution + 100 μL Catechol solution (1M) + 1 mL phosphate buffer; pH =7.0, ^2^10 mL.

sample solution + 100 μL dimethylgloyoxime solution (0.1 M) + 0.5 ml NH_4_Cl pH = 9.5, ^3^10 mL.

sample solution + 1 mL ammonium acetate buffer; pH =4.6.

### Statistical analysis

SPSS statistical package (Window version 18) and software Excel 2007 are used for data analysis. The analysis of the experimental data was carried out by using one-way ANOVA, Pearson correlation matrix, Cluster Analysis, Principal Component Analysis (PCA) and Factor Analysis (FA) methods [28,29]. Pearson correlation matrix shows a probable common source of the pollutants. Cluster analysis is used for dividing the studied metal ions into the similar classes with respect to their normalized concentration level. PCA is designed to transform the original variables into new, uncorrelated variables (axes), called the principal components. Factor Analysis is similar to Principal Component Analysis method except for the preparation of the observed correlation matrix for extraction and the underlying theory [30].

The one-way ANOVA method allows testing the significant difference of the means. For this test each sampling location was selected as a group and its heavy metal concentration as the corresponding variable. The ANOVA test requires three assumptions, i.e. the random behavior of the occurrence, the homogeneity of variance and the normal distribution behavior of the metal ions in the sample stations. These were tested by using Runs test, Levene statistic and the K-S (Kolmogorov-Smirnov) methods, respectively. It is noteworthy that instead of the ANOVA test, one can use the Kruskal-Wallis test. The latter is a non-parametric test without requirements announced for the ANOVA test [28,29]. In this work both of the methods were tested for a comparison.

The bivariate correlation procedure computes the pair wise associations for a set of variables and displays the results in a matrix. It is useful for determining the strength and direction of the association between two variables. The correlation coefficients computed by bivariate correlation procedure lay in the range −1 (for the cases in which a perfect negative relationship exists) to +1 (for a perfect positive relationship). A value of 0 indicates there is no linear relationship among the variables. For normally distributed variables, the Pearson method can be used to calculate the correlation coefficient. For normally distributed variables, the Pearson correlation was used for bivariate correlation, otherwise non-parametric Spearman method was applied.

Cluster analysis is a method for dividing a group of metals into classes so that similar metals, with respect to variable space, are in the same class. In fact, the groups are not known prior to applying this mathematical analysis and no assumption is made about the distribution of the variables [28,29].

The major objective of FA is to reduce the contribution of less significant variables to simplify even more of the data structure given by PCA. This goal can be achieved by rotating the axis defined by PCA and constructing new variables, also called Varifactors [31]. PCA reduces the dimensionality of data by a linear combination of original data to generate new latent variables which are orthogonal and uncorrelated to each other [32]. The major objective of FA is to reduce the contribution of less significant variables to simplify even more of the data structure coming from PCA. All significance statements reported in this study are at the P < 0.05 level.

## Results

### Extent of heavy metals contamination

The results of analysis of target metal ions i.e. Fe, Co, Ni, Zn, Cd and Pb in samples from 23 studied wells are given in Table 3. It is noteworthy that the reported values are based on three replicate determinations. Table 4 is prepared in order to give a simple comprehensive interpretation on the obtained data, and to compare the concentration of the studied metals in the samples with the MCL values reported by WHO and ISIRI. The results show that Fe, Co, Ni, Cu, Zn, Cd and Pb are detected in 100%, 47.8%, 78.3%, 100%, 100%, 65.2% and 100% of the samples, respectively. The concentration of metals (in μg /L) in the samples were found in the range of 75.90 -339.75 for Fe; ND (not detected) -99.82 for Co; ND −84.15 for Ni; 6.59-65.31 for Cu; 27.79 -2227.80 for Zn; ND −14.87 for Cd; and 0.74 -12.45 for Pb.

**Table 3:** Metal contents in water samples (μg/ L) from the wells

**Sample**	**Fe**	**Co**	**Ni**	**Cu**	**Zn**	**Cd**	**Pb**
W1	129.30 ± 10.91	9.91 ± 0.91	84.15 ± 10.84	44.39 ± 0.26	196.95 ± 11.40	4.55 ± 0.25	11.78 ±2.76
W2	339.75 ± 39.77	60.96 ± 1.95	52.24 ± 4.46	26.25 ± 2.03	169.51 ± 6.68	14.87 ± 0.86	6.10 ± 0.54
W3	143.99 ± 20.16	11.07 ± 1.15	41.59 ± 1.46	15.27 ± 1.44	140.53 ± 13.55	0.68 ± 0.06	1.26 ± 0.64
W4	228.71 ± 4.50	3.51 ± 0.82	42.98 ± 2.41	18.39 ± 1.46	460.19 ± 26.21	1.29 ± 0.45	1.67 ± 0.78
W5	194.11 ± 18.49	1.25 ± 0.15	21.82 ± 1.61	16.65 ± 1.05	353.92 ± 59.79	ND	2.63 ± 0.28
W6	109.83 ± 16.98	ND^2^	20.09 ± 1.23	8.83 ± 0.27	232.38 ± 41.84	ND	1.76 ± 0.42
W7	146.51 ± 3.63	0.43 ± 0.02	14.12 ± 0.91	15.71 ± 1.93	582.66 ± 61.54	3.55 ± 0.37	5.46 ± 0.48
W8	116.20 ± 9.88	ND	ND	27.71 ± 1.90	96.54 ± 6.23	ND	2.51 ± 0.16
W9	288.97 ± 16.40	1.89 ± 0.31	12.15 ± 0.44	65.31 ± 6.00	541.43 ± 22.53	3.41 ± 0.34	12.45 ± 0.73
W10	302.70 ± 20.74	2.24 ± 0.15	6.13 ± 0.22	35.81 ± 3.41	60.36 ± 6.74	0.36 ± 0.05	6.52 ± 0.31
W11	124.80 ± 7.37	ND	5.20 ± 0.21	12.06 ± 0.67	205.36 ± 12.64	ND	2.04 ± 0.19
W12	144.21 ± 15.68	99.82 ± 9.64	9.55 ± 0.41	21.96 ± 0.92	84.54 ± 6.23	0.62 ± 0.20	5.45 ± 0.38
W13	132.58 ± 6.49	ND	ND	9.84 ± 1.11	49.26 ± 2.11	1.23 ± 0.23	3.25 ± 0.24
W14	308.73 ± 22.94	ND	12.21 ± 1.11	13.79 ± 1.09	43.68 ± 2.15	0.83 ± 0.11	6.71 ± 0.24
W15	120.64 ± 24.25	1.52 ± 0.27	8.04 ± 0.28	60.77 ± 3.10	113.99 ± 7.12	ND	6.56 ± 0.31
W16	75.90 ± 10.80	3.19 ± 0.35	7.68 ± 0.47	31.95 ± 2.47	90.04 ± 7.71	ND	6.60 ± 0.24
W17	245.96 ± 29.36	ND	7.21 ± 0.24	21.52 ± 1.66	115.69 ± 15.27	1.43 ± 0.36	3.31 ± 0.27
W18	179.67 ± 22.70	ND	6.37 ± 0.27	8.22 ± 0.72	31.72 ± 4.87	0.73 ± 0.05	0.91 ± 0.07
W19	101.49 ± 6.39	ND	ND	24.87 ± 0.12	27.79 ± 1.51	0.59 ± 0.06	0.74 ± 0.21
W20	84.60 ± 12.10	ND	ND	28.17 ± 1.02	133.82 ± 15.59	0.15 ± 0.06	4.60 ± 0.22
W21	115.60 ± 32.23	ND	ND	6.59 ± 0.92	65.99 ± 4.21	2.25 ± 0.39	2.25 ± 0.32
W22	137.38 ± 23.88	ND	78.66 ± 6.60	15.02 ± 2.10	2227.80 ± 145.12	ND	5.26 ± 0.15
W23	113.57 ± 6.72	ND	5.57 ± 0.85	34.84 ± 3.75	73.67 ± 10.12	ND	2.56 ± 0.66

^1^Avearage of three determinations.

^2^Not Detected.

**Table 4:** Summery statistics of heavy metal content in water samples (μg/ L) analysis

	**Fe**	**Co**	**Ni**	**Cu**	**Zn**	**Cd**	**Pb**
Detected (%)	100	47	78	100	100	65	100
Min. of the detected concentration	75.90	ND^1^	ND^1^	6.59	27.79	ND^1^	0.74
Max. of the detected concentration	339.75	99.82	84.15	65.31	2227.80	14.87	12.45
Mean of the detected concentration	168.92	17.80	24.21	24.52	265.12	2.44	4.45
Standard deviation ^2^	77.95	32.33	25.21	15.50	456.66	3.68	3.14
MCL (based on WHO)	300.00^3^	-	70.00	1000	3000	3.00	10.00
Percentage of samples containing metals > WHO (%)	13.04	-	8.70	0	0	17.39	8.70
MCL (based on ISIRI) ^3^	-	-	70.00	1000	3000	3.00	10.00
Percentage of samples containing metals > ISIRI (%)	-	-	8.70	0	0	17.39	8.70

^1^Not Detected.

^2^Standard deviation for heavy metal concentration in all samples.

^3^Institute of standards and industrial research of Iran, 1997.

### Comparison of the concentration of heavy metals

In order to deduce the frequencies of the concentration of each metal in the samples, the Chi-Square test was applied [29]. Here, the frequency means the number of times a given range of concentrations occurs, and the Chi-Square test is used to examine whether the observed frequencies differ significantly from those which would be expected on the null hypothesis. This test indicates that there is no significant difference between observed frequencies of the heavy metals.

The random and normal distribution assumptions were checked by Runs and K–S methods, respectively. Another requirement for applying the ANOVA test is that the variances of the groups are equivalent. Based on the statistically verification done by Levene test, the homogeneity of variance was found to be significant for the samples (Levene statistic = 5.696, P < 0.001). Although the Levene statistic parameter rejects the null hypothesis, as the group variances are equal the ANOVA test can be yet used. Alternatively, the homogeneity and normal distribution in the data can be achieved by transforming the obtained data to another mathematically presentation which lowers the difference between the data. This can be achieved for example by using the logarithmic form of data. In addition, one can use a non-parametric test. This type of tests does not require to a homogeneity assumption.

The ANOVA method was used under two conditions. In fact, although the homogeneity of the data was not shown, ANOVA was applied to the data. In addition, by transforming the data as their logarithmic form, homogeneity in the observed data was achieved. The non-parametric method Kruskal-Wallis is based on ranks of the data variances. This method was used for the same scope as ANOVA. Both parametric and non-parametric methods used for comparison of the concentrations of heavy metals among sampling sites show a statistically significant difference depending on sampling locations.

### Bivariate correlations of investigated heavy metals

To deduce the probable common source of metals in water samples, the bivariate correlation procedure was used (Table 5). This procedure computes the pair wise associations for a set of metals and displays the results as a matrix. It is useful for determining the value of association of the investigated metals. Because, obtained data was not normally distributed, Spearman method was applied.

**Table 5:** Spearman correlation coefficient (r) of heavy metals in the sampling stations

	**Fe**	**Co**	**Ni**	**Cu**	**Zn**	**Cd**	**Pb**	**DO**
Co	0.019							
Ni	0.129	0.342						
Cu	0.007	0.008	−0.025					
Zn	0.121	**−0.433**^**a**^	**0.593**^**b**^	0.084				
Cd	0.291	0.027	**0.679**^**b**^	0.046	**0.551**^**b**^			
Pb	**0.283**^**a**^	−0.054	0.135	**0.583**^**b**^	0.168	**0.374**^**a**^		
DO	−0.079	0.273	**−0.681**^**b**^	−0.043	**−0.518**^**a**^	−0.436	−0.250	
Depth	**0.516**^**a**^	0.519	−0.258	0.003	−0.133	0.155	0.084	0.136
Dist. Ind.	0.266	0.027	0.234	0.029	0.080	0.261	0.241	0.063

Correlation is significant (a) at the 0.05 level and (b) at the 0.01 level.

### Classification of the investigated heavy metals by cluster analysis

**Figure 2:**
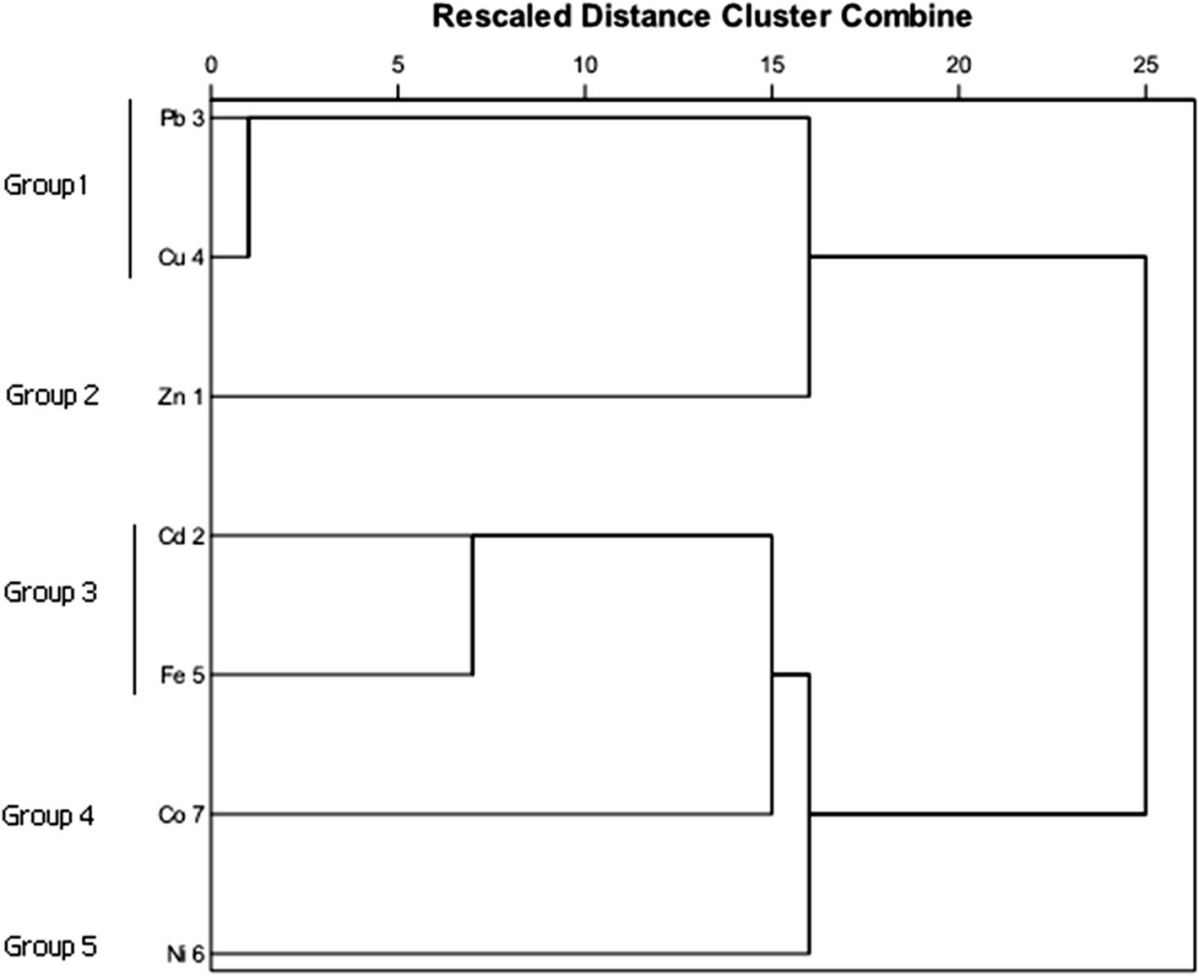
Dendrogram of heavy metal concentrations of water samples.

Cluster analysis grouped the studied heavy metals into clusters (called groups in this study) on the basis of similarities within a group and dissimilarities between different groups. CA was performed on the data using Ward method and squared Euclidean distance. A dendrogram was produced by cluster analysis, shown in Figure 2. Seven studied heavy metals were classified into five groups based on spatial similarities and dissimilarities.

### Principal component analysis and factor analysis

PCA reduces the dimensionality of data by a linear combination of original data to generate new latent variables which are orthogonal and uncorrelated to each other [32]. Prior to PCA and FA analysis, the raw data was commonly normalized to avoid misclassifications due to the different order of magnitude and range of variation of the analytical parameters [30]. The rotation of the principal components was executed by the Varimax method with Kaiser normalization.

Four principal components are obtained for heavy metals through FA performed on the PCA. This indicates that four main controlling factors influenced the quality of surface water in the study area. Corresponding components, variable loadings, and the variances are presented in Table 6. Only PCs with eigenvalues greater than 1 were considered. PCA of the whole data set yielded 4 data sets explaining 88.92% of the total variance. First component which explained 32.02% of the total variance is correlated with Pb and Cu. The second component is due to Zn and Co. The third component is a location for only Ni metal. The latest extracted factor is related to Fe and Cd.

**Table 6:** **Rotated component matrix of four-factor model**^**a**^

	**Component**			
	**1**	**2**	**3**	**4**
Fe	0.195	0.036	0.267	**0.881**
Co	−0.129	**−0.867**	0.047	0.142
Ni	0.044	−0.015	**−0.974**	−0.027
Cu	**0.960**	0.151	0.070	0.144
Zn	0.047	**0.858**	0.088	0.078
Cd	0.042	−0.140	−0.504	**0.808**
Pb	**0.961**	0.047	−0.123	0.078
Eigen value	2.241	1.708	1.216	1.059
% of total variance	32.020	24.405	17.366	15.132
% Cumulative of variance	32.020	56.425	73.791	88.924

^a^Extraction method: Principal component analysis. Rotation method: Varimax with Kaiser Normalization. Rotation converged in 5 iterations.

## Discussion

According to results mentioned in Table 4, all of the samples contained Co, Cu and Zn inferior to the values specified by related MCLs. In contrast, in 13.0% and 8.7% of the samples the amounts of Fe and Ni, respectively, were above WHO MCLs. The amount of cadmium found in 34.8% of the samples was lower than the detection limit of the DPP method, but 17.4% contained the metal ion superior than the ISIRI and WHO MCL. This is of concern because cadmium has carcinogenic properties as well as a long biological half life leading to chronic effects as a result of accumulation in liver and renal cortex. It can also cause kidney damage as well as producing acute health effects resulting from over exposure to high concentrations [20].

Due to possible long term effects of chronic exposure, the presence of lead in drinking water is crucially important for public concern. Although all of the samples included this metal, 8.7% of them contained lead ions above the levels proposed by WHO and ISIRI MCL. Overall average concentration of heavy metals in water samples varies as Zn > Fe > Cu ≈ Ni > Co > Pb > Cd. The results reveal that the amount of heavy metals depends on the sampling locations.

As shown in Table 5, a close relationship between the couples Fe/Pb, Cu/Pb, Cd/Pb, Co/Zn, Ni/Zn, Ni/Cd and Zn/Cd states a probable common source of the couples. A further statistical investigation was performed by testing the correlation between the determined concentration of heavy metals and the distance of the sampling site from NILZ Company. The calculated correlation (Cu/Pb and Zn/Cd) can confirm the significant effect of NILZ Company activities as a main source of heavy metal contamination observed in the investigated groundwater samples. In addition, close correlation between Fe and depth of the wells (0.52) suggests that this metal is totally of pedo-geochemical source leached from the upper soil layers.

Cluster analysis allows identification of five clusters or groups of associated metals (Figure 2). On the basis of similarities found for group 1 (Pb, Cu), one can suggest the anthropogenic origin of the contamination sources. The presence of iron in group 3 (Cd, Fe) notifies, probably, mixed anthropogenic and pedo-geochemical source of the metals presented in this group. Therefore Zn, Co and Ni were located in single member groups.

Also according to Table 6, Component 1 is attributed to lead and copper with positive sign. These elements are important byproducts of lead industries indicating its anthropogenic sources. Component 2 reveals 24.4% of the total variances are positively loaded with Zn and negatively loaded with Co. Component 3 shows that 17.4% of the total variance is positively loaded with Ni and it can be represented by oil industries activities near the NILZ Company. Component 4 explains 15.1% of the total variance, is positively loaded with Cd and Fe.

**Figure 3:**
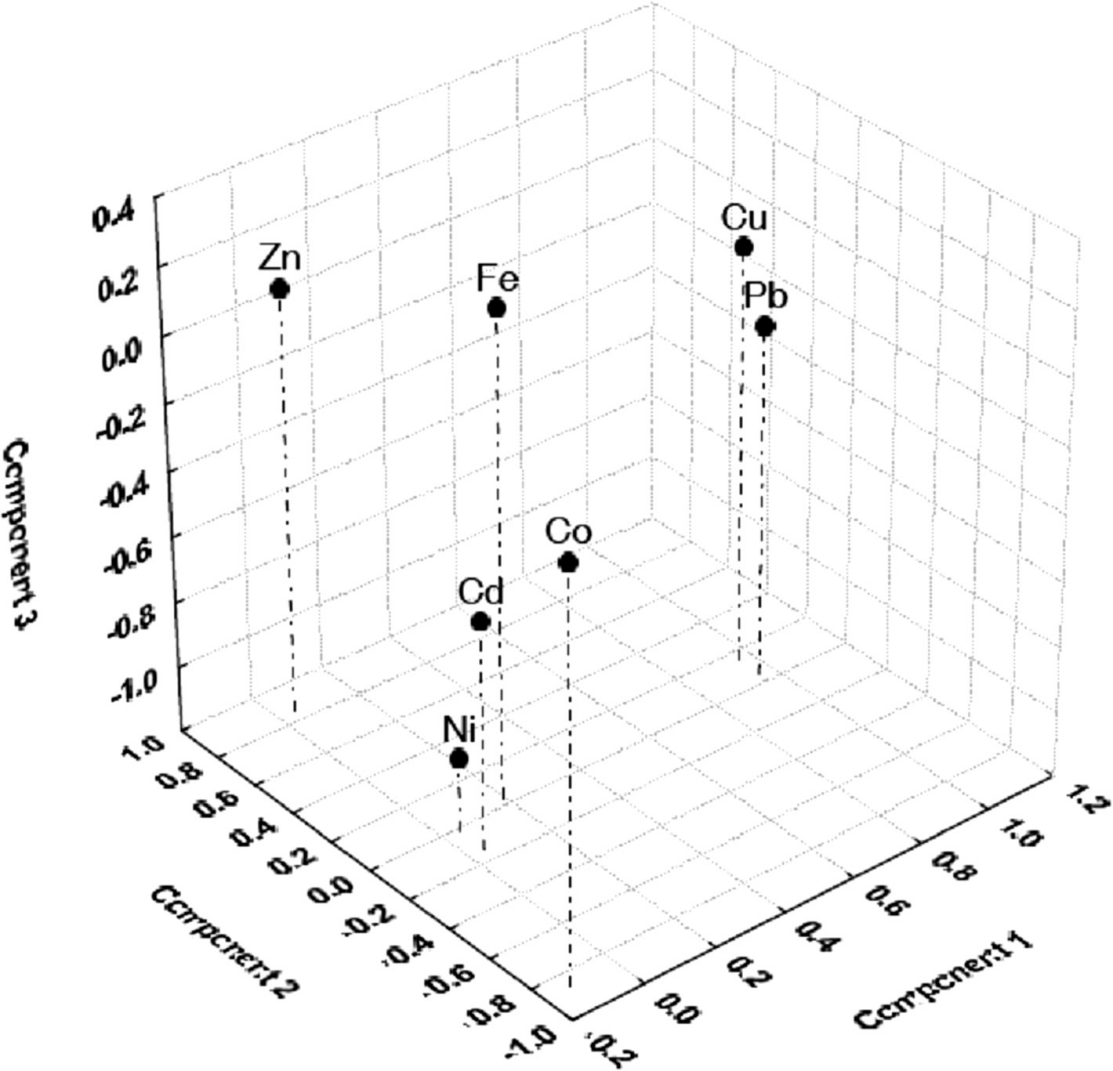
Component plot in rotated space for heavy metals (Factor loadings, factor 1 vs. factor 2 vs. factor 3, Rotation: varimax normalized, extraction: principal components).

The heavy metal grouping has been explored in the plot of the first three principal components generated from these parameters (Figure 3). The low correlation found for the studied metal ions in the four components defined by FA, suggests both anthropogenic and pedo-geochemical sources for the metal contaminations.

## Conclusion

Overally, the present study has shown that the groundwater source within radius of 5 km from National Iranian Lead and Zinc Company (NILZ) in Bonab Industrial Estate (Zanjan province-Iran) is contaminated by iron, cobalt, nickel, copper, zinc, cadmium and lead. This can be considered as a menace for people who daily intake the corresponding waters, planted vegetables and food crops irrigated by the same water source. The higher health risk comes from those elements which are present at higher levels than announced by WHO and ISIRI notably lead, nickel and cadmium. Multivariate statistical techniques have shown correlations and similarities among the investigated heavy metals and classification of these ion groups. Cluster analysis has identified five clusters among the heavy metals. The statistical investigations reveal the pollution sources influencing water quality in the study area as anthropogenic (with a very high contribution of NILZ Company) and pedo-geochemical for Fe, Cu. The results suggest a significant risk to the population of Zanjan city and its neighborhoods given the toxicity of the studied metals and the fact that this aquifer by far is the main source of their drinking water and irrigation. This study has also highlighted the need for further research and regular monitoring, in order to determine the permitted levels of metals in the studied aquifer.

## Competing interests

The authors declare that they have no competing interests.

## Authors’ contributions

This work is part of the PhD thesis of AAZ where MRY and AHP, supervised the thesis, suggested the problem, participated in determination of sample points, sample preparation procedure, and wrote and edited the manuscript. All authors read and approved the final manuscript.
